# Identifying barriers in the malaria control policymaking process in East Africa: insights from stakeholders and a structured literature review

**DOI:** 10.1186/s12889-015-2183-6

**Published:** 2015-09-04

**Authors:** Christopher Paul, Randall Kramer, Adriane Lesser, Clifford Mutero, Marie Lynn Miranda, Katherine Dickinson

**Affiliations:** Nicholas School of the Environment & Duke Global Health Institute, Duke University, Durham, NC USA; Duke Global Health Institute, Duke University, Durham, NC USA; Centre for Sustainable Malaria Control and School of Health Systems and Public Health, University of Pretoria, Pretoria, South Africa, & International Centre of Insect Physiology and Ecology, Nairobi, Kenya; Rice University, Houston, TX USA; National Center for Atmospheric Research, Boulder, CO USA

## Abstract

**Background:**

The complexity of malaria and public health policy responses presents social, financial, cultural, and institutional barriers to policymaking at multiple stages in the policy process. These barriers reduce the effectiveness of health policy in achieving national goals.

**Methods:**

We conducted a structured literature review to characterize malaria policy barriers, and we engaged stakeholders through surveys and workshops in Kenya, Tanzania, and Uganda. We compared common barriers presented in the scientific literature to barriers reported by malaria policy stakeholders.

**Results:**

The barriers identified in the structured literature review differ from those described in policymaker surveys. The malaria policy literature emphasizes barriers in the implementation stage of policymaking such as those posed by health systems and specific intervention tools. Stakeholder responses placed greater emphasis on the political nature of policymaking, the disconnect between research and policymaking, and the need for better intersectoral collaboration.

**Conclusions:**

Identifying barriers to effective malaria control activities provides opportunities to improve health and other outcomes. Such barriers can occur at multiple stages and scales. Employing a stakeholder - designed decision tool framework has the potential to improve existing policies and ultimately the functioning of malaria related institutions. Furthermore, improved coordination between malaria research and policymaking would improve the quality and efficiency of interventions leading to better population health.

## Introduction

Malaria is a complex and pernicious disease caused by a plasmodium parasite and transmitted by mosquitoes, resulting in severe morbidity and mortality globally. Sub-Saharan Africa alone experiences around one - half million deaths annually [1]. Malaria is a complex vector-borne disease in that the parasite has a multi-stage lifecycle with two hosts: mosquitoes and humans. Malaria control thus engages a multifaceted response of both prevention, as through the use of bed nets, and treatment, such as use of antimalarial drugs. Throughout the policymaking process, policymakers face many barriers to applying available resources and knowledge towards identifying and implementing effective interventions to combat malaria.

Given the complex policy process for malaria, how should researchers and policymakers characterize barriers to the development and implementation of effective policy? The aim of this paper is to identify policy barriers in the malaria policy process, specifically in East Africa, relying on data from both the scholarly literature and stakeholders, with the larger goal of shedding light on how barriers may affect the public health policy process more broadly. Identifying policy barriers ultimately presents opportunities to address them.

In public policy analysis, the policy process is popularly described by Jann and Wegrich as: agenda-setting, policy formulation, decision making, implementation, and evaluation [2]. Within this process, there has been limited attention to systematically defining barriers that may impede progress at each stage. This paper makes three key contributions to the literature on conceptual understandings of decision making and the policy process: 1) we develop a conceptual framework of barriers in a policy process, 2) we investigate barriers in the malaria policy process using a structured literature review and policymaker input, and 3) we apply this framework to a specific example of malaria policymaking in East Africa, the Malaria Decision Analysis Support Tool (MDAST). We build upon prior work with MDAST which describes the potential role of decision tools [3] and the importance of stakeholder enagement [4].

The paper continues as follows. First, in the background, we further develop the concept of barriers in the policymaking process, and describe examples in malaria policy specifically. We then introduce the methods and research activities used for this project in conjunction with the development of the MDAST Project. Specifically, we use two methods: a structured literature review, in comparison to malaria policy stakeholder perspectives, as collected in surveys and workshops. In the results and discussion, we summarize the findings of the structured literature review and stakeholder activities. In the conclusions, we discuss the application of structured decision-making processes, such as decision tools, in response to barriers.

## Background

**Fig. 1:**
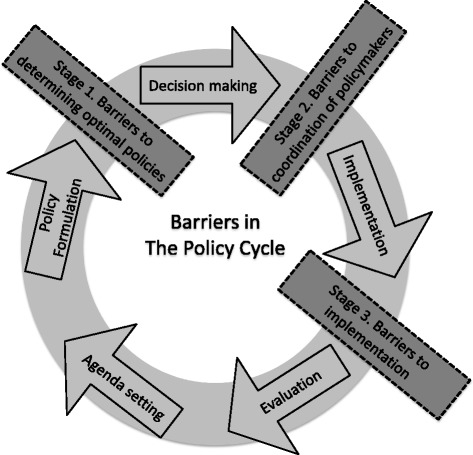
Barriers in the Policy Process

The policy process of agenda-setting, policy formulation, decision making, implementation, and evaluation is depicted in Fig. 1 [2]. Barriers, which we define as conditions that prevent progress towards stated policy goals, exist throughout the policy process and in a wide variety of dimensions, including social, financial, cultural, and institutional. We focus on barriers in three stages of the policy process: 1) determining effective policies, which occur between policy formulation and decision making; 2) coordinating policymakers around the policies, in advance of implementation; and 3) implementing identified policies, as labeled in Fig. 1. In practice, these stages might not always be distinct. We focus on these particular barriers, in the policy formulation, coordination, and implementation stages, to gain a greater understanding of what prevents policymakers from fully utilizing available assets and knowledge to achieve effective policy. These programmatic barriers can be distinguished from the agenda-setting stages of policymaking (e.g., setting targets and objectives), which we do not attempt to address here.

Policymakers in malaria control encounter a range of barriers in the process of determining, coordinating around, and implementing effective policies. First, in the stage of determining policies, barriers include the need to assimilate diverse information and objectives. The coordination stage is complicated by the complex nature of the disease and the variety of possible interventions. Malaria control generally involves multiple agencies and organizations across governance scales, and there is a need for coordination among actors in the policy process [3]. For example, while a ministry of health manages treatment of patients, vector control (e.g. household insecticide spraying) may fall within the mandate of a ministry of environment or agriculture. Even if malaria control is coordinated by a single agency, managing the many activities of different divisions is difficult [5]. Agencies may operate on varying geographic scales and with different time frames and malaria control strategies. International organizations and donor agencies are particularly influential when determining the scope and type of many malaria control activities [6]. Finally, barriers in the implementation stage include logistical complexities, as well as lack of capacity, resources, and sustained political willpower. Throughout the policy process, policymakers need access to information that specifically addresses or helps to overcome potential barriers, not just more information overall [7]. An “evidence-based policymaking” approach is needed to be able to be adaptive to context [8].

We evaluate the role of malaria policymaking barriers using the experience of the development of the Malaria Decision Analysis Support Tool (MDAST), a project in East Africa supported by the Global Environment Facility, United Nations Environment Programme and World Health Organization. MDAST is a tool and forum to promote evidence-based, multi-sector malaria control policymaking, with Kenya, Tanzania, and Uganda serving as pilots for other malaria-prone countries [3]. The decision tool models potential malaria control interventions, the simultaneous effects of these potential actions on human health, environmental, and economic outcomes, and provides a forum for policymakers to jointly consider these options [4]. Properly implemented, structured decision processes with tools such as MDAST can help policymakers identify and address barriers. This paper integrates input from stakeholders specifically on barriers with data from scientific literature to improve the understanding of barriers, and particularly in relation to decision tools.

## Methods

In this paper, we use two methods to understand barriers in the malaria policy process: a structured literature review and stakeholder input in the form of workshops and surveys that were conducted as part of the MDAST project. Our approach primarily relies on qualitative content analysis, with limited descriptive quantitative analysis, to triangulate key characteristics of policy barriers [9].

### Structured literature review

In order to better characterize the nature of barriers in malaria policymaking and inform the baseline development of MDAST, a structured literature review from the previous 15 years (1996–2011) on the term ‘malaria policy barriers’ was conducted using Google Scholar in July 2011. This period approximately represents modern international malaria policy with the development of what became the Roll Back Malaria partnership in 1998, until the initiation of the MDAST project. Additionally, the landscape of malaria control was fundamentally altered during this period by the emergence of major funding sources such as the President’s Malaria Initiative and The Global Fund to Fight AIDS, Tuberculosis and Malaria and the accompanying steep rise in international funding for malaria control (from under 100 million USD in 2000 to 1.6 billion USD in 2011) [1].

A structured literature review systematically identifies prominent themes and concepts with content analysis [10]. This is a different approach than meta-analysis common in the biomedical literature, such as Cochrane Reviews, which attempt to synthesize a numerical summary result across multiple studies [11]. Google Scholar (http://scholar.google.com) is an appropriate tool for this approach given the wide scope of disciplines that may document malaria interventions and Google’s prominence as a search engine for anyone, including policymakers, seeking information. Google’s proprietary natural language search algorithm indexes and analyzes results from across all available online academic databases, and produces equivalent results to other databases for meta-analysis [12].

The Google Scholar search using the term ‘malaria policy barriers’ identified approximately 16,700 potential papers. The first 200 listings, as ranked in order of relevance by the search algorithm, were evaluated for their relevance to policymaking barriers. Articles were excluded if they were: a) not on the topic of malaria (75 articles); b) on the topic of malaria but did not include a discussion on policy (42 articles); c) if they had a specific geographic focus outside of sub-Saharan Africa, given our focus on stakeholders in East Africa (14 articles); or d) not peer-reviewed (4 articles). Using these criteria, sixty-six of the 200 articles (33 %) were deemed relevant for analysis and were subsequently classified by topical areas.

After the selection of relevant articles, we identified 17 general qualitative categories of barriers, which we group as follows: behavior and individual characteristics, health systems, social and structural features, policy processes, and environment. Each article was then reviewed independently by two researchers for a second time and assigned to one or more category. The researchers then reviewed discrepancies in their classifications to generate a final list of relevant articles and coding for descriptive analysis of major themes and barrier type. The results from the qualitative content analysis are summarized below, and served as references in the development of MDAST.

### Stakeholder survey and workshops

As an additional part of the development of MDAST, malaria policy stakeholders from Kenya, Tanzania, and Uganda participated in a survey and workshops in July and August 2010 on malaria policymaking activities in their country [13]. The survey respondents were drawn from a non-random purposeful sample of key stakeholders selected by lead MDAST project collaborators in each country (Division of Malaria Control in Kenya, Vector Control Division in Uganda, and the National Institute for Medical Research in Tanzania). The multimodal (email and hardcopy) survey targeted individuals in ministries, NGOs, universities and research institutes whose decisions and actions have malaria policy implications.

Stakeholder workshops were held in each of the three MDAST project countries during August 2010 with a subsection of the survey participants. Participants came from a range of sectors and agencies, including from government ministries of health, environment and agriculture. The workshops were designed around stakeholder-based analysis of the malaria policy process in their respective countries. Specifically, stakeholders were asked to develop graphical representations of decision processes (“influence diagrams”), showing malaria policy interventions, and identifying potential barriers to these interventions [14]. The discussion sessions during the workshop, including the evaluation of participant-generated influence diagrams, were structured as focus group discussions [15]. We elicited participants’ perspectives on malaria intervention decision-making, policymaking challenges in their countries, and how a decision tool could enhance policy. Workshop facilitators led identification and discussion of common themes in the final section of each workshop. Facilitators (namely, four of the authors) summarized the information and opinions provided by stakeholders during each of these sessions both by country and overall, and these summaries were evaluated to determine prominent themes and trends, as presented in the results below.

Ethical approval of the study was received in advance from the Institutional Review Board at Duke University (Protocol A0009); an exemption was granted for the specified data collection methods and procedures and informed written consent was therefore neither required nor obtained. The data from the survey and workshop are fully anonymous. The survey and stakeholder activities were conducted in coordination with official agencies in each country.

## Results

### Structured literature review

The 66 relevant papers identified through the Google Scholar search and subsequent analysis were assigned to one or more categories as tabulated in Table 1. The majority of the citations referred to barriers that would affect the implementation of specific policies, which would be implementation stage three barriers. Articles which discuss the policy process (29 % of articles) address stage one policy determinant barriers. Stage two coordination barriers are relevant to the 22 % of articles coded for health systems. Observations for specific types of barriers in the literature are summarized by category below, with citations given as examples. A complete list of the literature review is available from the authors upon request.

**Table 1:** Barrier classification categories

Category (non-exclusive)	Count (of 66 total)	Percent of total addressing this barrier
Behavior and individual characteristics		
- Adherence: Barriers to proper adherence by households to malaria control regimes	16	24.2 %
- Information and Education: Barriers arising due to lack of education, including about the causes of malaria and means to treat malaria.	8	12.1 %
- Wealth: Barriers arising due to poverty or limited resources	9	13.6 %
Health systems		
- Access: Ability of populations to access healthcare system	24	36.4 %
- Availability: Availability of drugs and other malaria control measures.	13	19.7 %
- Funding and financing: Barriers arising from limited funding and financing for malaria control	10	15.2 %
- Health Systems: Organizational and institutional capacity of health systems	22	33.3 %
- Laboratory infrastructure: Barriers arising from the limitations of laboratory equipment	4	6.1 %
- Research programming and priorities: Barriers to appropriate research programs and priorities	3	4.5 %
Social and structural features		
- Culture: Barriers arising from cultural norms which conflict with malaria control policy	12	18.2 %
- Gender: Barriers arising from gender norms and disparities	6	9.1 %
- Equity: Barriers arising from structural inequity	16	24.2 %
Policy process and government		
- Policy: Barriers arising from policy and policymaking constraints, institutional fragmentation	19	28.8 %
- Taxes and tariffs on malaria control goods: Barriers of taxes and tariffs on goods for malaria control	1	1.5 %
Environment		
- Pesticide toxicity and contamination: Barriers arising from concern of pesticide toxicity and contamination	1	1.5 %
- Resistance: Barriers arising from the development of resistance by the malaria parasite or mosquito to treatment	4	6.1 %
- Seasonality: Barriers arising from the seasonal/cyclical nature of malaria	3	4.5 %

#### Behavior and individual characteristics

Sixteen articles discuss barriers to achieving appropriate adherence to treatment at the individual level, which is most relevant to barriers in implementation, while four articles discuss resistance directly. Individual and community education levels affect malaria control outcomes which is discussed in eight (12.1 %) articles. Nine articles (13.6 %) dealt with the role of household wealth as a barrier to malaria treatment or control. In addition to primary care, associated costs such as transport and time to seek treatment are also a major restriction on the poor for malaria control [16].

#### Health systems

Twenty-four of the 66 (36 %) relevant articles in this search describe some element of access to care. Access to healthcare systems is determined by location of clinics and available transport, capacity of the system, and education about the health system. Poor populations tend to have less access to care, for example as reported in a study on malaria treatment in Kenya [17]. Geographic and financial limitations to accessing care are likely correlated, compounded by the impact such factors have on the establishment of health systems [18]. Private vendors of malaria treatment and control generally provide greater geographic access but are unlikely to conform to policy norms or drug standards, and may be untrained to provide malaria treatment guidance.

Approximately 33 % of the articles included in the review refer to the quality of the health systems as a major factor in malaria control, and represent stage two barriers of coordination. For example, health workers in Uganda need significant training themselves in order to convince the Ugandan public of the importance of malaria treatment and adherence [19]. Drug demand must be anticipated and drugs properly stocked [17].

#### Social and structural features

Cultural norms and beliefs play an important role in the acceptability of malaria control measures. For example, a study from Tanzania reports the belief that intermittent preventive treatment (IPT) in pregnancy weakens the mother and causes poor birth outcomes [16]. In a systematic review of qualitative studies in Africa, a general lack of understanding about the disease remains a major barrier to prevention [20]. Four of the articles discuss how conditions of equity, or inequity, affect the outcomes of malaria control policies. Huge gaps of access exist for poor, rural, and vulnerable populations [17, 21, 22]. The average effect of an intervention may hide inadequate outcomes for lower resource households.

Fifteen articles (21 %) discuss IPT as the primary intervention. Malaria treatment and prevention in pregnant women is a component of prenatal care in some countries, yet overall implementation of IPT is limited [18]. Six studies discuss the role of gender specifically, reporting the extra barriers women often face to receiving care, such as the need to find childcare. Special populations need to be targeted with specific education campaigns, as in the case of IPT [23]. Many of the studies also suggest the importance of educating men, who are often the household decision maker, to the particular issue of preventing malaria in pregnancy and childhood [24].

#### Policy process and government

Malaria control poses a serious challenge for the organizational capacity in most countries. For example, taxes on malaria control materials not only increase costs, but involve another layer of government before reaching project implementation [25]. Determination and implementation of appropriate drug regimes for treatment require capacity and training at all levels, from national drug approval to appropriate diagnosis and prescription at the clinical level [26].

#### Environment

The toxicity of pesticides used in malaria control is a commonly expressed concern in malaria control policy, though this literature review included only one paper discussing the topic [27]. Three articles identified the cyclical and seasonal nature of malaria transmission as an impediment to control efforts, which are further complicated by development of resistance by both mosquitoes and the malaria parasite. Additionally, climate introduces uncertainty and may present a significant barrier to control efforts [17].

### Stakeholder activities

Our stakeholder survey and workshop results represent a broad spectrum of policymakers in East Africa. We surveyed 97 individuals (Kenya = 33, Uganda = 33, Tanzania = 31). Forty-nine percent of survey respondents work for government, 22 % for universities or research institutions, 9 % for NGOs, and 20 % for other types of organizations. Seventy-one percent of the respondents primarily work in the health sector, 10 % work in the agricultural sector, 7 % in the environment sector, and 5 % in the education sector (the remaining proportion reported working in other sectors). Each workshop had representatives from the country’s malaria control program, the WHO, and civil society, including researchers. Twenty-two stakeholders participated in Uganda workshop, 21 in Tanzania, and 15 in Kenya.

Overall financial resources are a major determinant in decision making, according to data from the survey and workshops, which primarily is a stage three barrier to implementation. The survey also indicated a greater need for research to inform the policy process, and a desire for a less politically-driven agenda in the malaria control process [13]. The survey results were echoed in comments during the discussion in the stakeholder workshops. For example a participant stated, “good technical decisions can be reversed by political forces”, a theme which was common from all three stakeholder workshops. Strategic integration of research efforts was a common theme across all of the workshops. Participants in Tanzania and Uganda specifically noted the importance of intersectoral collaboration in both research and malaria control implementation. In Kenya, stakeholder workshop discussions indicated the importance of aligning priorities of research organizations with national health research needs.

When survey participants were asked for a free response regarding “any challenges, obstacles, or other issues involved in formulating a national malaria control strategy and combating malaria in your country”, 58 % of respondents (49 of *n* = 85 complete responses) indicated concerns about stage three barriers to implementation, as compared to 47 % mentioning stage one barriers to determining optimal policies, and 44 % mentioning stage two barriers to coordination of policymakers. For example, respondents mentioning barriers to coordination and implementation gave comments including “funding cycles do not line up with malaria cycles, such as the onset of the rainy season”.

Workshop participants indicated that development of vector and parasite resistance presents a significant risk and that environmental impacts of malaria control should be given greater importance than they are currently. In the Uganda workshop, participants from the agricultural sector expressed particular interest in an integrated vector management approach. Participants expressed specific concern that insecticide treated nets may become a much less effective tool as the mosquitoes develop resistance to the insecticides used, noting how agricultural sector practices also contribute to pesticide resistance. Participants in Tanzania expressed concern about the potential impacts on trade and tourism of the re-introduction of DDT, a controversial chemical historically used in malaria control.

During the workshops, stakeholders highlighted the need for improved collaboration among researchers and decision makers in different sectors, institutional settings, and levels of government. In Kenya, stakeholders noted the need for a sustained venue for bringing researchers and policymakers together (*e.g.*, a national health research conference), also noting that researchers need to make their findings more accessible and meaningful to policymakers. Politicians may be crisis-oriented or face pressures incompatible with the research process, such as short time frames and demand for direct assistance programs. Participants in Uganda indicated that in the past national leaders enacted policy decisions without engagement of the malaria policymaking community. Stakeholders in Tanzania noted that different levels of decision-making faced different sets of internal and external motivations for engaging in specific forms of malaria control. At the national level, decision makers respond to donor preferences, whereas at the local level, the reputation of the implementing organization is a key concern. Participants in Tanzania urged more community involvement in malaria policy.

## Discussion

The two methods of literature review and stakeholder input identify a variety of important barriers in malaria policymaking. Notably, the literature review and the stakeholder input emphasize different types of barriers. The literature predominantly discusses barriers in the implementation stage of malaria policymaking, addressing specific challenges such as those in health systems and intervention tools. In contrast, the stakeholder responses highlight the political nature of policymaking, and the disconnect between research and policymaking, with less emphasis on specific intervention barriers. Ultimately, recognition of barriers to policy and implementation is insufficient; policymakers must have processes to identify and enact solutions across multiple scales and stakeholders.

A structured decision-making tool and a process to acknowledge barriers provides policymakers with the opportunity and tools to consider tradeoffs among a wide range of policy solutions. However, improving policy processes across sectors requires significant investment and coordination. Aid agencies and national governments must devote resources to the coordination and empowerment of policymakers at all levels, and disseminate these policies to the organizations, agencies, and staff who implement programs and malaria interventions at the community level.

Decision tools such as MDAST are designed to assist policymakers in considering multiple interventions and impacts in concert. Improving decision-making includes increased transparency, available and accessible information, and an improved dialogue between policymakers and implementers from different sectors. Employing a comprehensive decision framework provides a structured approach to the identification of barriers, illuminating opportunities to improve existing policies and ultimately the functioning of institutions. Yet, policy leaders must provide mechanisms for better coordination among the multiple actors involved. A structured decision-making process can prompt cross-sector discussion about barriers to improved malaria policymaking. Nonetheless, a decision tool is insufficient to alter outcomes. The potential benefits of decision tools will ultimately be realized only if stakeholders have opportunities for increased communication and collaboration in malaria policymaking.

The approach of this paper has some limitations. The qualitative content analysis of the literature review and the stakeholder input, while useful for revealing key themes and forms of barriers, do not generate specific quantitative parameters for use in decision tools or other contexts. Decision tools such as MDAST heavily rely upon meta-analyses, which estimate general effects of interventions, such as indoor residual spraying or bed nets, to use in modeling [28, 29]. As another limitation, the stakeholders, while representing a broad sample of malaria policymakers in each country, were not randomly selected, and thus results do not necessarily reflect the average perception by policymakers in those countries. Finally, the policymaking process for any issue is complex and political, and efforts to inform decision tools and processes should acknowledge that policymaking involves a variety of actors and forces.

## Conclusions

This paper discusses and identifies barriers in the malaria policy process, which by definition prevent the full functioning of policies and programs. The identification of barriers to effective malaria control activities can productively be viewed as a set of opportunities to improve health and other outcomes. As evidenced by the literature review and stakeholder activities, such barriers can occur at multiple stages and scales. Also, while stakeholders expressed some similar concerns across the three countries, much of the literature and stakeholders responses emphasize local context. The process of employing a comprehensive decision tool framework provides a structured approach to identifying barriers and the associated opportunities to improve the functioning of existing programs and policies. This, in turn, should lead to improved outcomes by enabling existing institutions to be more effective. By considering barriers across the whole spectrum of malaria control, national-level policy makers can better allocate resources. International agencies can support policy makers to these ends by providing tools, forums, and resources to consider a broader policy process. National policymakers rarely have the opportunity to meaningfully coordinate across agencies working in malaria control whereby they can eliminate barriers by maximizing combined resources. Decision tools and stakeholder engagement incorporating understanding of barriers, such as with MDAST, can be designed in a way as to mitigate those barriers to policymaking. Future work should evaluate how use of decision tools reduces barriers in practice.

## Abbreviations

IPT: Intermittent Preventive Treatment
MDAST: Malaria Decision Analysis Support Tool
